# The effect of form and method of flavoring on microbiota of olive oil

**DOI:** 10.1002/fsn3.3664

**Published:** 2023-09-08

**Authors:** Tereza Kacalova, Alzbeta Jarosova, Olga Cwikova, Gabriela Franke, Tereza Soumarova

**Affiliations:** ^1^ Department of Food Technology Mendel University in Brno Brno Czechia

**Keywords:** flavouring, microbiological analysis, olive oil

## Abstract

Flavoring olive oil is an increasing trend in olive oil processing. Growing consumer interest in flavored olive oils by natural material brings the need to evaluate the key limiting factors which is its microbiological stability. The present research compares the microbiological quality of olive oil flavored by 3 flavors (rosemary, garlic, and lemon), prepared by 3 methods to determine changes during storage. The comprehensive microbiological analyses (total number of microorganisms [TCM], anaerobic sporulates, yeasts, molds, bacteria of the family *Enterobacteriaceae*, bacteria of the genus *Salmonella* spp., *Clostridium botulinum*, and lactic acid bacteria) were conducted during 12 months of storage. The best results in TCM were observed in the oil flavored by fresh garlic (0.24 log CFU/mL). The highest counts of anaerobic sporulates were detected in the dried rosemary olive oil (1.10 log CFU/mL). The flavoring materials have significantly higher counts of microorganism than flavored oils (*p* < .05). The obtained results demonstrated that microorganisms are capable to survive in flavored olive oil and the method of flavoring can affect their growth in a selective way according to the chemical characteristics.

## Graphical abstract

The article compares the microbiological quality of flavoured olive oils. 3 flavourinf forms were used in this study and 3 methods of flavouring. The total number of microorganisms (TCM), anaerobic sporulates, yeasts, molds, bacteria of the familly Enterobacteriaceae, bacteria of the genus *Salmonella* spp., *Clostridium botulinum*, and lactic acid bacteria were examined and the effect of flavouring on the growth of microorganisms was observed.

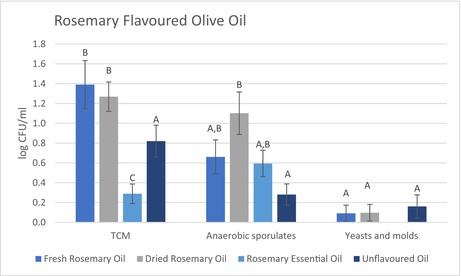

## 1 INTRODUCTION

Various herbs, spices, and essential oil are used to extend the shelf life utilizing growth inhibition effect on foodborne pathogens in foodstuffs (Rockwell & Raw, 1979). Wide range of flavoring methods are used: flavoring by natural material, essential oil or addition of the material during pressing process (Li et al., 2014; Sena‐Moreno et al., 2018). From the results of research conducted by Benkhoud et al. (2022), the essential oils could be used effectively for olive oil flavoring as it has good sensory attributes, oxidative stability during storage, no significant effects on the fatty acid profiles, and nutritional quality index.

Compounds naturally present in the olive oil (carotenoids, phenols, flavonoids) contribute to its antimicrobial, antiviral, antiparasitic, and other health protective properties which help to maintain populations of microorganisms within limits of detection (Tripoli et al., 2005; Waterman & Lockwood, 2007).

Lipid oxidation and microbial changes are the main reasons for the deterioration of food quality, food safety, and shelf‐life. The presence of pathogens in the foodstuff can be cause of serious diseases leading to death. Gram‐positive *Staphylococcus aureus*, *Listeria monocytogenes* and Gram‐negative *Salmonella typhimurium*, *Escherichia coli*, and *Campylobacter jejuni* are among the most common bacteria present in the foodstuff (Görner & Valík, 2004).

Virgin and extra virgin olive oil are unique among edible oils, they contain a small amount of water forming small droplets. Microbes may be present in this aqueous phase, but the physical size of the droplets generally limits their amount (Ciafardini & Zullo, 2002). Olive oil has been considered a food that does not provide a suitable environment for the growth of microorganisms. However, recent research has shown that olive oil can be contaminated with microorganisms (especially yeasts) that are able to affect the oil's properties (Ciafardini et al., 2017).

Antimicrobial properties of various oils were tested (Karaosmanoglu et al., 2010; Keceli & Robinson, 2002; Medina et al., 2006; Zullo et al., 2018). Virgin olive oils were more effective than olive pomace oils. Virgin olive oils were less effective against *Escherichia coli* and *Shigella sonnei* than against other bacteria. Content of phenols (hydroxytyrosol, tyrosol) can delay the growth of certain bacteria, especially *Streptococcus thermophilus*. Coliform bacteria can survive and reproduce in virgin olive oil containing low levels of phenolic compounds.

Ciafardini et al. (2013) conducted a study during the oil sedimentation to determine whether the hydrolysis of oleuropein that occurs during the decantation and storage of olive oil is catalyzed by olive enzymes or microorganisms present on the olive fruit. Microscopic observation showed the presence of microorganisms in water droplets and sediments. The yeasts *Saccharomyces cerevisiae* and *Candida wickerhamii* were the most abundant in olive oil samples, while bacteria and mold were not found. The study revealed that the oleuropein present in olive oil can be hydrolyzed by β‐glucosidase from yeast, the filtration was suggested despite a significant reduction in polar phenols. Recent studies have shown that *Candida parapsillosis* can survive in olive oil for a very long time, exceeding 24 months. Mold belonging to the genus *Aspergillus* have been also observed in olive oil. Giavalisco et al. (2023) observed the microbiota of olive oil and its positive and negative effect on olive oil. Strains belonging to *Candida boidinii, Lachancea fermentati, Nakazawaea molendinolei, N. wickerhamii*, and *Schwanniomyces polymorphus* were observed. Yeasts limited the acidity rise in olive oils, but increased the parameters related to oxidative phenomena, causing a declassification of EVOOs. According to Ciafardini et al. (2013), yeasts (*Saccharomyces cerevisiae* and *Candida wickerhamii*) are among the most frequently occurring microorganisms in olive oils, and the presence of fungi (genus Aspergillus) has also been observed.

Olive oil blending is widely used for the production of sufficient quantities of olive oil with a balanced taste to meet the demands of the global market. In the study (Ciafardini & Zullo, 2002) was demonstrated that the microbiota survives well in the monovarietal olive oil but not in the blended olive oils after 1 month of storage. Several oil‐borne yeasts derived from healthy olives has positive effect as they improve some sensory characteristics of the oil. Some yeasts are derived from damaged olive drupes and can impair the quality of olive oil under favorable conditions, such as high‐water content and low phenolic concentration.

Olives are source of natural non‐pathogenic epiphytic bacteria, but can be contaminated during growth, harvesting, transport, and further processing in mills by pathogens from animal and human sources. Contamination can be caused by treating the soil with organic fertilizers, such as sewage sludge, manure, and irrigation water (Zullo et al., 2018).

When the garlic containing spores of *C. botulinum* is merged with oil in anaerobic environment, the spore germination and the growth of microorganisms promote. For this reason, herbs and vegetables in the oil must be properly treated to prevent botulism poisoning. Commercial mixtures of garlic in oil are acidified to prevent the growth of bacteria. These products can then be stored safely at room temperature, otherwise should be refrigerated (Nummer et al., 2011; Raab, 2015). Botulism, the paralytic disease caused by a toxin produced by the bacteria *Clostridium botulinum*, outbreaks after the consumption of garlic in olive oil stored at room temperature was reported (Morse et al., 1990).

A range of methods to identify strains of microorganisms were used in recent studies. The identification by screening a high number of colonies grown on a specific chromogenic medium (Ciafardini & Zullo, 2002) or method when protein is extracted and directly analyzed via MALDI‐TOF MS resulting in a mass spectral profile, which is characteristic for the considered organism (Gantzias et al., 2020).

The aim of the present research was to determine and compare the numbers of microorganisms in flavored and unflavored oil and to assess the effect of flavoring method on the microorganisms in the oils. Also, to assess the effect of flavoring component addition on microbiological quality of the olive oil.

## 2 MATERIALS AND METHODS

### 2.1 Material

The virgin filtered olive oil was obtained from olive fruit (*Olea europea* L.) variety Aayrouni, handpicked from the olive orchards located in the region North Lebanon. Aayrouni is an old variety still found in some ancient Lebanese groves. It is mainly cultivated in the North, and the Mount Lebanon regions. This cultivar is characterized by the high oil content of fruit, ranging from 34% to 37% expressed on fresh weight. This determines their use mainly for the oil production (Chehade et al., 2016). A two‐phase continuous extraction system was used. Garlic, rosemary, and lemon were used for flavoring (different kind of flavoring agent). Rosemary plants (*Rosmarinus Officinalis* L.) were purchased from local gardening store (Brno, Czechia). The garlic cloves (*Allium sativum* L.) were obtained from a family farm (Moravia, Czechia), and organic lemons (*Citrus limon* L.) were purchased at local store (Brno, Czechia). Olive oil was obtained from Lebanese Genco Olive Oil (Baassir, Lebanon). The acidification treatment by citric acid solution (Abo et al., 2014) of the garlic and rosemary was carried out, as storage of low‐acid plant material in oil is known botulism risk.

Samples were prepared in 3 different methods (forms) of flavoring as following: fresh and dry material have same origin and is from the same batch, fresh material was processed accordingly (acidified, dried), fresh garlic 4.7 g/100 mL of olive oil, 1.3 g/100 mL of dried garlic, and 50 μL/100 mL (0.05% w/v) of garlic essential oil (Zan Aromi, Brno, Czechia), fresh rosemary 2 g/100 mL, dried rosemary 0.7 g/100 mL, rosemary essential oil (Zan Aromi, Brno, Czechia) 50 μL/100 mL (0.05% w/v), fresh lemon peel 4 g/100 mL, dried lemon peel 1 g/100 mL, and lemon essential oil (Aromatics, Czechia) 150 μL/100 mL (0.15% w/v) as described in Table 1. The oil jars were stored for 12 months at room temperature (20 ± 1°C) in a dark and dry area until analysis. The flavoring materials and the unflavored oil were analyzed. The following analyses were carried out every 3 months.

**TABLE 1:** Description of oil samples included in the experiment. Total number of 258 samples (*n* = 258) were analyzed.

Sample flavoring	Designation	Number of samples	T0	T3	T6	T9	T12
Fresh rosemary	RF	*n* = 24		*n* = 6	*n* = 6	*n* = 6	*n* = 6
Dried rosemary	RD	*n* = 18		*n* = 6	*n* = 6	*n* = 6	
Rosemary essential oil	REO	*n* = 18		*n* = 6	*n* = 6	*n* = 6	
Fresh garlic	GF	*n* = 24		*n* = 6	*n* = 6	*n* = 6	*n* = 6
Dried garlic	GD	*n* = 18		*n* = 6	*n* = 6	*n* = 6	
Garlic essential oil	GEO	*n* = 24		*n* = 6	*n* = 6	*n* = 6	*n* = 6
Unflavored oil 1 control group	N1	*n* = 30	*n* = 6	*n* = 6	*n* = 6	*n* = 6	*n* = 6
Fresh lemon	LF	*n* = 24		*n* = 6	*n* = 6	*n* = 6	*n* = 6
Dried lemon	LD	*n* = 24		*n* = 6	*n* = 6	*n* = 6	*n* = 6
Lemon essential oil	LEO	*n* = 24		*n* = 6	*n* = 6	*n* = 6	*n* = 6
Unflavored oil 2 control group	N2	*n* = 30	*n* = 6	*n* = 6	*n* = 6	*n* = 6	*n* = 6

#### 2.1.1 Sample preparation

Samples were stored for a year. They have been analyzed in 3 months intervals. First analysis T0 of unflavored oils and flavoring materials were analyzed separately according to Witkowska et al. (2011).

In case of evidence of *Salmonella* spp., a 25 g of sample was added in the homogenization bag with 225 mL peptone water (Interscience), the bags were left for 24 h at 42°C in a thermostat (Schoeller instruments) to allow the pre‐multiplication. In the case of detection of other microorganisms, 10 g of the sample and 90 mL of physiological saline were added to the homogenization bag, homogenized, and a series of decimal dilutions were made. In the determination of sporulating anaerobic microorganisms, the tubes with appropriate dilutions were heated in a water bath (Julabo TW 20 Schoeller instruments) for 20 min at 80°C to eliminate vegetative forms of spore‐forming bacteria and other microorganisms.

#### 2.1.2 Preparation of culture medium

A mixture of nutrient medium was weighed into a glass bottle and flooded with distilled water in a certain ratio according to the manufacturer's instructions. The culture medium was mixed and sterilized at a steam sterilizer at 121°C. Yolk emulsion and CBI supplement (*Clostridium botulinum* isolation) were added to sterile agar to determine *Clostridium botulinum*. A 1 mL of the sample was pipetted (Thermo scientific) into the petri dishes, poured with the appropriate nutrient medium (soil pouring method), and mixed in a circular motion. After solidification, the plates were placed in a thermostat (Thermo Scientific).

#### 2.1.3 Cultivation

Petri dishes with inoculated medium were placed in a thermostat under certain conditions suitable for the growth of the microorganisms. Once the incubation period had elapsed, the plates were removed from the thermostat and evaluated by counting characteristic colonies (POL‐EKO APARATUR). Results were expressed in units of CFU/mL.

### 2.2 Determination of the total number of microorganisms

The determination of the total number of microorganisms was performed according to the standard ČSN EN ISO 4833‐1: 2014. A 1 mL of inoculum of respective dilution was inoculated into a petri dish and flooded with Plate Count Agar (PCA). Incubation under aerobic conditions was carried out for 72 h at 30°C, and for anaerobic sporulates for 72 h at 37°C. Determination of the total number of microorganisms includes mesophilic aerobic and facultative anaerobic microorganisms that grow on nutrient media under aerobic and anaerobic conditions. All grown colonies on agar were counted for evaluation.

### 2.3 Determination of the number of anaerobic sporulating microorganisms

The determination of the number of anaerobic sporulating microorganisms was performed according to EN ISO 4833‐1: 2014 (vegetative forms of microorganisms were inactivated at 85°C/10 min; incubation was carried out under anaerobic conditions in an anaerostat).

### 2.4 Determination of yeasts and molds

The determination of yeasts and molds was performed according to the ČSN ISO 21527‐2: 2009. From the prepared dilution, 1 mL of inoculum was taken, inoculated onto a petri dish with agar. Incubation of the petri dishes with Dichloran Rose Bengale Chloramphenicol (DRBC) agar was carried out for 2–5 days at 25°C under aerobic conditions. The yeasts form rounded colonies of a slightly pinkish color on the agar. The molds form flat colonies of several cm in size with different coloration (blue, green, white). Typical colonies were counted during the evaluation.

### 2.5 Determination of bacteria Enterobacteriaceae

For the determination of *Enterobaccteriaceae* bacteria, VRBG soil (Violet Red Bile Glucose) was used according to ČSN EN ISO 21528‐2 (2021). The appropriate amount of sample (1 mL) was inoculated onto a plate and covered with VRBG agar. Incubation was carried out for 24 h at 37°C under aerobic conditions. Confirmation: proof of oxidase negative, glucose fermentation positive.

### 2.6 Determination of *Salmonella*

*Salmonellae* were determined according to the standard ČSN EN ISO 6579‐1: 2020. The determination was performed in two steps. First, the medium was pre‐multiplied in a thermostat for 20 h and then multiplied on medium. From the pre‐propagated medium, 0.1 mL of inoculum was taken and inoculated onto a petri dish with solidified nutrient medium. The inoculum was then spread with sterile forceps. The petri dishes were incubated in a thermostat for 24 h at 37°C. For the determination of bacteria of the genus *Salmonella* spp. petri dishes with already solidified Salmonella IRIS agar were inoculated directly.

### 2.7 Determination of clostridium botulinum

*Clostridium botulinum* was determined according to the standard ČSN 560090: 1987. Yolk emulsion and CBI supplement were added to the sterilized agar, then 1 mL of the dilution of 10 was taken, inoculated onto a petri dish and covered with agar. Cultivation was performed in a thermostat at 37°C for 48 h under anaerobic conditions.

### 2.8 Determination of lactic acid bacteria

MRS soil (De Man, Rogosa, and Sharpe) was used for the detection of lactic acid bacteria in accordance with ČSN ISO 15214: 2000. 1 mL of the respective dilution was taken, inoculated onto a plate and covered with agar. Cultivation took place on MRS soil for 3 days at 30°C. The evaluation consisted of counting typical colonies that are round and regular, either white or colorless.

### 2.9 Statistical analysis

Statistical evaluation was performed using Statistica 14 and Microsoft Excel 2019. For better clarity, the results were converted to log CFU/mL. Basic statistical characteristics were calculated (mean, standard error of the mean), and one‐factor ANOVA analyses of variance, including Duncan's post hoc test, was used to compare individual files.

## 3 RESULTS AND DISCUSSION

### 3.1 Effect of flavoring method

**FIGURE 1:**
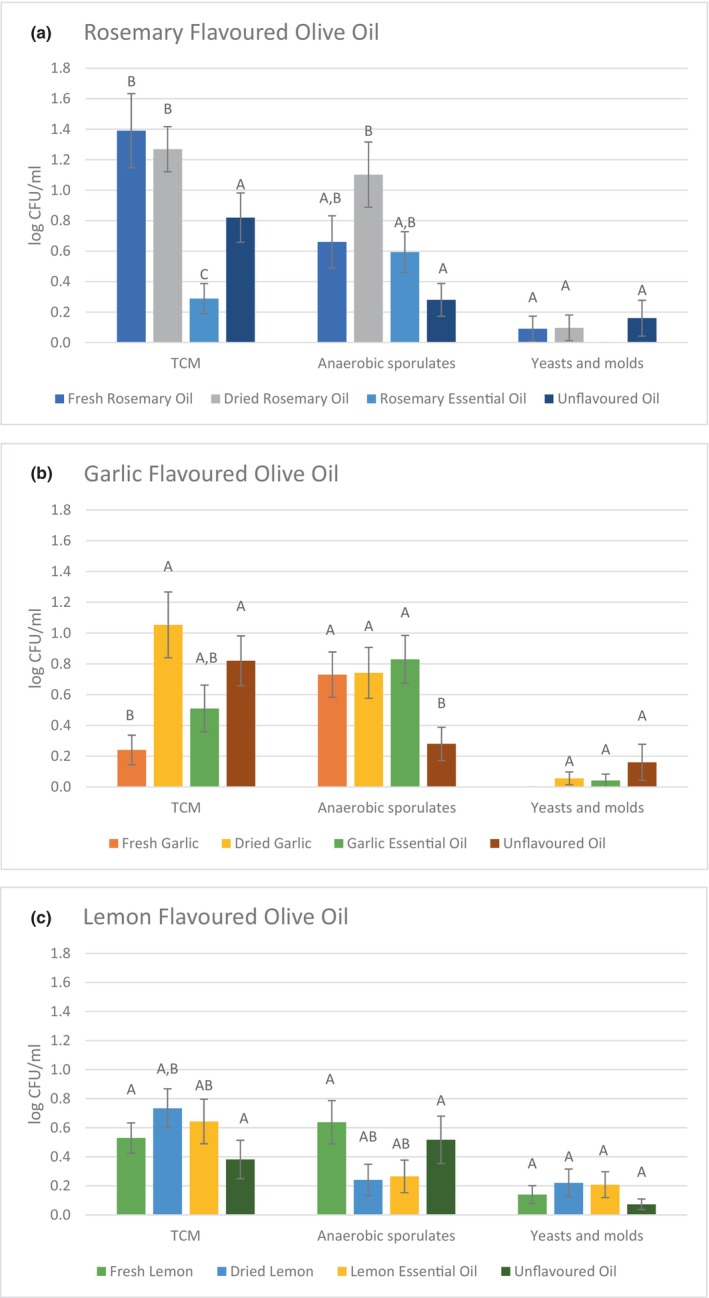
Comparison of the total number of microorganisms, the number of sporulating anaerobic micro‐organisms, the number of yeasts and molds (log CFU/mL) in: (a) rosemary flavored oils; (b) garlic flavored oil; (c) lemon flavored oil prepared by different methods and in unflavored oil. Different letters within the study set (flavoring method) indicated significant difference (*p* < .05), (*n* = 102).

The effect of the flavoring method on the number of microorganisms in oils flavored with rosemary, garlic, and lemon was evaluated (Figure 1). Data were evaluated regardless of storage time.

The total count of microorganisms was lower (*p* < .05) in the oil flavored with rosemary essential oil (0.29 log CFU/mL) compared to the unflavored oil (0.82 log CFU/mL). The highest (*p* < .05) count was found for the oil flavored with fresh rosemary (1.39 log CFU/mL) as shown in Figure 1a which confirms the hypothesis that using raw material for flavoring increase the number of microorganisms. Olivas‐Méndez et al. (2022) observed the effect of rosemary essential oil to the growth of microorganisms in beef burgers. The significant decrease of TCM, yeasts, and molds comparing to the control was observed. The same trend was observed in the present study. The highest counts (*p* < .05) of anaerobic sporulating microorganisms were in the oil flavored with dried rosemary (1.10 log CFU/mL) comparing to the unflavored oil (0.28 log CFU/mL).

The oil flavored with fresh garlic contained the lowest (*p* < .05) counts of total number of microorganisms 0.24 CFU/mL (Figure 1b) which confirms the hypothesis that addition of flavoring material with antimicrobial properties decrease numbers of microorganisms. Garlic flavored oil clearly differentiated from other types of flavors, in similar study (Ciafardini et al., 2004) was observed complete absence of any microorganism, and low content of molds was observed. The numbers of anaerobic microorganisms in all garlic flavored oils ranged from 0.73 to 0.83 log CFU/mL were significantly higher (*p* < .05) than unflavored oil.

The lemon flavored oils are compared in Figure 1c. The total numbers of microorganisms in the oils flavored with fresh, dried lemon, essential oil, and the unflavored oil ranged from 0.38 log CFU/mL (unflavored oil) to 0.73 log CFU (oil with the addition of dried lemon). There was no difference in total microorganism counts between the oils tested (*p* > .05). Numbers of anaerobic sporulates were detected ranging from 0.24 log CFU/mL (oil flavored with dried lemon) to 0.64 log CFU/mL (oil flavored with fresh lemon). There was no difference (*p* > .05) in the numbers of anaerobic sporulating microorganisms between the oils we analyzed.

When comparing oils flavored with different forms of lemon and unflavored oil, no differences in yeast and molds counts were observed (*p* > .05). Counts ranged from 0.07 log CFU/mL (unflavored oil) to 0.22 log CFU/mL (oil flavored with dried lemon). The method of flavoring did not affect the presence of microorganisms, this hypothesis was not confirmed in lemon flavored oils.

The work of Zanoni et al. (2015) focused on the comparison of microbiological quality in extracted and filtered olive oil. In the extracted one, yeasts were present in 1.65 log CFU/mL, while in the filtered one, yeasts were detected <1 log CFU/mL, suggesting that fungal contamination in unfiltered oil may be affected by the hygienic level of the olives. The total number of microorganisms was not detected at in the filtered oil, in the extracted oil was 2.45 log CFU/mL. The filtered oils in the present study contained a total count of 0.82 log CFU/mL, 0.38 log CFU respectively. The count of yeasts and molds 0.16 log CFU/mL, 0.07 log CFU respectively. TCM were slightly higher in the oil we tested, while yeast counts were comparable.

Ciafardini et al. (2004) found the presence of total microorganisms, yeasts and fungi in oregano‐flavored olive oil. The number of molds was in the range of 4 log CFU mL and TCM 5.21 log CFU/mL, these values are higher comparing to flavored oils studied which might be attributed to the procedure prior flavoring (washing, acidification). Garlic flavored olive oil had low counts of microorganisms, which may be due to the fact that some garlic components have various antimicrobial effects that may affect the growth of microorganisms. This effect was also confirmed in our study.

Tripoli et al. (2005) observed an antibacterial effect, when the flavoring treatment with mixture of parsley, fresh garlic and lemon efficiently inhibited the growth of bacteria not exceeding log 3 CFU/mL. Considering the upper value 7 log CFU/mL to be the microbiological limit of the acceptable quality of food (ICMSF, 1986).

The obtained data confirm the hypothesis according to which the different flavoring method influences the survival of the microorganisms by modifying the habitat. In fact, the results as a whole obtained from the subsequent microbiological analyses carried out during 12 months storage strongly confirm the hypothesis. According to our results the flavoring by essential oils did not significantly increase TCM in flavored oils as the manipulation with raw material is eliminated.

### 3.2 Effect of primary contamination

The flavoring materials were contaminated by various microorganisms. The microbiological analysis of flavoring material itself (fresh, dried, essential oil) was carried out and then added to the olive oil. Data were compared regardless of the time of sampling.

**FIGURE 2:**
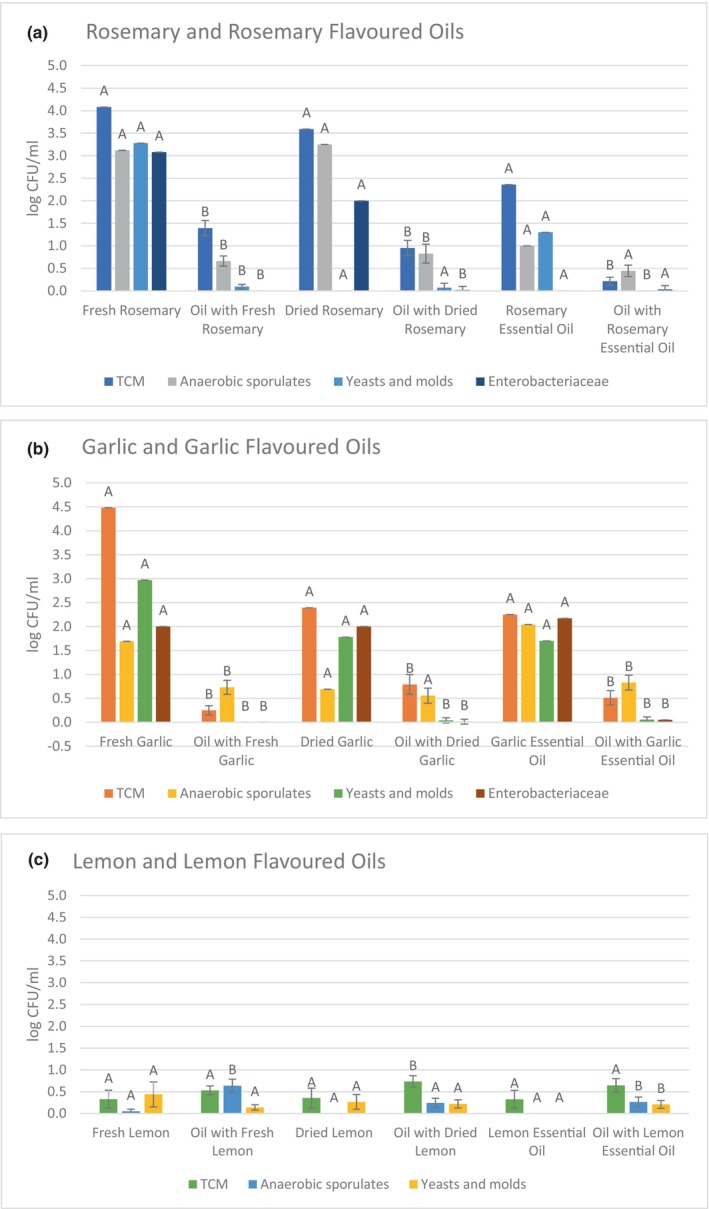
Comparison of the total count of microorganisms (TCM), the number of sporulating anaerobic microorganisms, the number of yeasts and molds, and Enterobacteriaceae bacteria (log CFU/mL) in (a) rosemary and rosemary flavored oils, (b) garlic and garlic flavored oil, and (c) lemon and lemon flavored oil. The averages indicated by different letters differ within the study set (*p* < .05).

There was a significant difference (*p* < .05) between the numbers of microorganisms in fresh rosemary and oil with added rosemary for all groups of microorganisms studied (Figure 2a). The mean numbers of microorganisms in the oils were always lower (*p* < .05) than in the flavoring material alone. While rosemary contained total counts of microorganisms ranging from 2.36 to 4.08 log CFU/mL, the oil with rosemary contained TCM ranging from 0.22 to 1.39 log CFU/mL. The numbers of anaerobic sporulates in rosemary were found to range from 1 to 3.25 log CFU/mL, and in the oil with added rosemary from 0.45 to 0.83 log CFU/mL. Yeasts and molds in fresh rosemary and rosemary essential oil reached values of 3.28 and 1.30 log CFU/mL, respectively, and were not detected in dried rosemary. Yeasts and molds were detected in fresh and dried rosemary oils at an average of 0.09 log CFU/mL. Bacteria of the *Enterobacteriaceae* family were detected only in fresh rosemary (3.08 log CFU/mL) and dried rosemary (2 log CFU/mL). Enterobacteriaceae were detected at 0.02 and 0.03 log CFU/mL in oils with dried rosemary and essential oil.

For all forms of garlic treatment, the counts of microorganisms were higher (*p* < .05) compared to oils samples (Figure 2b). Total counts of microorganisms in garlic flavoring materials were detected in the range of 2.25–4.48 log CFU/mL, while counts in garlic oils range between 0.25–0.79 log CFU/mL. Anaerobic sporulates were present in garlic oils within 0.55 log CFU/mL to 0.83 log CFU/mL, while in fresh garlic, dried garlic and garlic essential oil counts ranged from 0.69 log CFU/mL to 2.04 log CFU/mL. Yeasts and molds were recorded in all garlic flavoring material in the range of 1.70–2.97 log CFU/mL. In the oils with garlic, yeasts and molds were only present in the oils with dried garlic (0.04 log CFU/mL) and in the oils with garlic essential oil (0.04 log CFU/mL). Enterobacteriaceae family were present at oil with dried garlic 0.01 log CFU/mL, and oil with garlic essential oil 0.05 log CFU/mL. Bacteria of the Enterobacteriaceae family were detected in fresh garlic, dried garlic and garlic essential oil from 2.0 to 2.17 log CFU/mL.

Comparing counts of fresh lemon and oil with the addition of fresh lemon, the numbers of TCM, fungi and yeasts, respectively, were balanced (*p* > .05). The numbers of aerobic microorganisms were 0.33 log CFU/g in the fresh lemon and 0.53 log CFU/mL in the oil after its addition. As for molds and yeasts, the counts were 0.44 log CFU/g in fresh lemon and 0.14 log CFU/mL in oil. The counts of anaerobic sporulates in the fresh lemon were 0.05 log CFU/g, counts increased (*p* < .05) after the addition of the oil to 0.64 log CFU/mL. The increase might be caused by the unflavored olive oil, its values are TCM 0.38 CFU/mL, anaerobic sporulates 0.52 CFU/mL, and yeasts and molds reaching 0.07 CFU/mL.

The aerobic counts in dried lemon were 0.36 log CFU/g, while the counts of oil after its addition the increased (*p* > .05) to 0.73 log CFU/mL. The counts of anaerobic sporulates and fungi and yeasts were comparable in both dried lemon and oil after its addition (*p* > .05). The counts of fungi and yeasts were 0.22 log CFU/g in dried lemon and 0.27 log CFU/mL in oil with its addition. Higher yeasts and molds count 2.12 and 2.91 log CFU, respectively, were observed at commercial lemon flavored oil (Ciafardini et al., 2004). Anaerobic sporulates were not detected in the dried lemon, counts of 0.24 log CFU/mL were recorded in the oil.

The numbers of microorganisms of the observed groups were higher in olive oil with the addition of lemon essential oil, compared to essential oil. The numbers of aerobic microorganisms increased (*p* < .05) from 0.33 to 0.64 log CFU/g or mL in the case of the oil. Anaerobic sporulates and yeasts and molds were not detected in the essential oil, but counts were higher (*p* < .05) in the oil after its addition, with 0.27 log CFU/mL for anaerobic sporulates and 0.21 log CFU/mL for yeasts and molds. It can be assumed that the anaerobic sporulates originate solely from the extra‐virgin olive oil used in the experiment.

These findings were noted in researchers observing herbs and spices primary contamination, which are related to the grown, harvesting, and processing practice (Ankri & Mirelman, 1999; Ciafardini et al., 2004). From the comparison of data of microbiological analyses of rosemary and garlic flavored oils, it can be seen that ingredients with olive oil inhibit the survival of microorganisms. In flavored olive oil, the survival of the microorganism depends on the method of flavoring and olive oil used.

The hypothesis that addition of flavoring component affects the microbial quality of flavored oil was not confirmed. In the majority of flavored oil samples regardless of the flavoring component was no increase in counts of microorganisms The anaerobic environment and the antimicrobial properties of olive oil itself reduce the ability of growth of microorganism.

### 3.3 Determination of lactic acid bacteria, *Clostridium botulinum* and *Salmonella* spp.

Several cases of botulism have been associated with herbs stored in oil. Our research has focused on the determination of *Clostridium botulinum* in flavored oils. In the present study, neither lactic acid bacteria, *Salmonella* spp. nor *Clostridium botulinum* were detected during the entire storage period.

Medina et al. (2006) dealt with the antimicrobial activity of olive oil compared to other vegetable oils. The results revealed that olive fruit oils had a strong bactericidal effect against a wide range of microorganisms, and this effect was generally higher against Gram‐positive than Gram‐negative bacteria. Thus, olive oils exhibited bactericidal activity not only against harmful bacteria of the intestinal microbiome (*Clostridium perfringens* and *E. coli*) but also against beneficial microorganisms such as *Lactobacillus acidophilus* and *Bifidobacterium bifidum*. Otherwise, most of the foodborne pathogens tested (*Listeria monocytogenes*, *Staphylococcus aureus*, *Salmonella enterica*, *Yersinia* spp. and *Shigella sonnei*) did not survive after 1 h of contact with olive oils. In another study of Karaosmanoglu et al. (2010), it was observed the antimicrobial activity of extra virgin olive oil against three foodborne pathogenic bacteria *Escherichia coli*, *Listeria monocytogenes*, and Salmonella Enteritidis. Although all the extra virgin olive oils showed bactericidal activity, the individual phenolic compounds showed little antimicrobial activity. Moreover, the refined oil samples did not show any antimicrobial activity.

## 4 CONCLUSION

In conclusion, the present complex study has examined the effect of three flavoring methods (flavoring by fresh material, dry material, and essential oil) and storage time (3 months intervals for 12 months storage) on the microbiological quality of olive oil using three flavors (rosemary, garlic, and lemon). The parameters of interest were TCM, anaerobic sporulates, yeasts, molds and also counts of microorganisms typical for flavored olive oil (bacteria of the family *Enterobacteriaceae*, bacteria of the genus *Salmonella* spp., lactic acid bacteria), including toxin forming *Clostridium botulinum*. Due to the fact that the detailed production conditions of flavoring material were not possible to control, the effect of only one independent variable on the above‐mentioned parameters was considered: the method of flavoring. The flavoring method factor did not affect the count of yeasts and molds in the oil samples. The three tested flavored oils had different effects on counts of microorganisms: total counts of microorganisms and anaerobic sporulates (*p* < .05) were significantly affected by method of flavoring only at rosemary and garlic flavored oil groups. However, no clear trend was observed in lemon flavored oil either in the TCM or anaerobic sporulates. The method of flavoring affects the microorganism persistency, independently of the primary contamination. Such a characteristic can be particularly observed when using flavoring material with high antimicrobial activity. This confirmed the natural antimicrobial property of garlic and its positive impact on microbiological quality of flavored olive oils.

Significantly higher counts were observed in rosemary and garlic flavoring material more than the flavored oils themselves regardless of the flavoring method. The laboratory results of microbiological analyses carried out showed that microorganisms are capable of surviving in olive oils. The above‐mentioned results confirm the antibacterial properties of flavoring plants, herbs and spices. It also proves the ability of olive oil to maintain the growth of microorganisms in foodstuff due to its high content of polyphenolic compounds. So, from the viewpoint of flavored oil producers, the results of the present study indicate differences in microbiological quality parameters typical for flavored olive oils due to the method of flavoring.

## AUTHOR CONTRIBUTIONS

**Tereza Kacalova:** Data curation (equal); formal analysis (equal); investigation (equal); methodology (equal); validation (equal); visualization (lead); writing – original draft (lead). **Alzbeta Jarosova:** Methodology (supporting); supervision (equal); validation (equal); writing – review and editing (equal). **Olga Cwikova:** Data curation (equal); formal analysis (equal); investigation (equal); methodology (lead); supervision (equal); validation (equal); writing – review and editing (equal). **Gabriela Franke:** Supervision (supporting); validation (supporting); writing – review and editing (equal). **Tereza Soumarova:** Formal analysis (equal); investigation (equal); writing – original draft (equal).

## CONFLICT OF INTEREST STATEMENT

The authors declare that there is no conflict of interest.

## Data Availability

The data that support the findings of this study are available on request from the corresponding author.
